# A novel anatomically pre-contoured side-specific titanium plate versus the reconstruction plate for quadrilateral plate fractures of the acetabulum: a propensity-matched cohort study

**DOI:** 10.1186/s13018-020-01659-w

**Published:** 2020-05-14

**Authors:** Haiyang Wu, Ranran Shang, Ximing Liu, Chengjing Song, Yanzhao Chen, Xianhua Cai

**Affiliations:** 1 Department of Orthopaedic Surgery, General Hospital of Central Theater Command, Wuhan, 430070 Hubei China; 2 Southern Medical University, Guangzhou, 510515 Guangdong China

**Keywords:** Acetabular fracture, Quadrilateral, Fracture reduction, Fixation, Reconstruction plate, Models

## Abstract

**Background:**

Surgical treatment of acetabular fractures involving a quadrilateral plate is a challenge to orthopedic surgeons. We have developed a novel fixation technique using a specially shaped reconstruction plate combined with several buttress screws of a quadrilateral plate which was also called a dynamic anterior plate-screw system for quadrilateral plate (DAPSQ) to treat acetabular fractures involving quadrilateral plate since 2005 (RP group). And the long-term follow-up results have confirmed the effectiveness and safety of this technique. After 2016, standardized titanium plate (STP group) of DAPSQ have been designed and applied. The aim of the study was to compare the clinical efficacy of anatomical plate and the reconstruction plate of DAPSQ in the treatment of quadrilateral plate fractures.

**Methods:**

We led a propensity-matched cohort study of quadrilateral plate fractures. Twenty-two patients were included in the STP group during the inclusion period (2016–2018) and were matched to 22 cases in our database of the RP group (2008–2016). The primary outcome measures were the quality of reduction and functional outcomes. Intraoperative conditions were also compared.

**Results:**

Of these 22 consecutive patients in the STP group, the mean age was 46.7 years and the most common fracture pattern was a both-column fracture (12 cases, 54.5%) according to Letournel-Judet classification. The mean follow-up period was 23.1 months (range 12–37). There were no significant differences between the two groups with regard to the quality of reduction using the Matta radiological criteria and functional outcomes evaluated by the modified Merle d’Aubigné score (*P* > 0.05). Compared with the RP group, the STP group had a shorter operation time (245.1 min vs. 286.8 min, *P* = 0.020), less intraoperative blood loss (1136.4 mL vs. 1777.3 mL, *P* = 0.014), and transfusion (780.9 vs. 1256.8 mL, *P* = 0.035). The complication rate was 18.2% in the STP group, and there was no significant difference compared with the RP group (36.4%) (*P* > 0.05). None of the cases in the two groups had quadrilateral screws entering the hip or implant failure.

**Conclusions:**

The fixation of standardized titanium plate in quadrilateral plate fractures showed a similar result to the reconstruction plate, in terms of quality of reduction and functional outcome. The standardized titanium plate of DAPSQ has the advantages of a short operation time, less intraoperative bleeding, and blood transfusion, and it is worth further promotion and research.

## Background

With the development of industrialization and urbanization in China, high-energy injuries caused by traffic or construction accidents are gradually increasing, and the incidence of acetabular fractures is also increasing year by year [1]. In order to allow early mobilization, improve functional outcomes, and decrease the risk of post-traumatic arthritis, operative reduction and internal fixation (ORIF) has been considered as the gold standard for the treatment of displaced acetabular fractures [2, 3]. Letournel-Judet classification is one of the most widely used clinical classifications of acetabular fractures. According to the acetabular double-column theory, acetabular fractures are divided into 10 categories, including 5 simple fractures and 5 complex fractures [2]. It is worth noting that in addition to the simple anterior or posterior wall fractures, the other 8 types of acetabular fractures may involve an important anatomical structure called a quadrilateral plate. The quadrilateral plate is located on the inner surface of the acetabulum, deep in position, thin in the bone, and adjacent to a large number of the peripheral nerves and blood vessels. When suffering from high-energy trauma, medial subluxation of the femoral head and dome impaction, as well as a high degree of comminution fractures are common in this area. Chang et al. [4] noted that the reduction of the quadrilateral plate fracture played an important role in the surgical results of acetabular fractures, and more than 80% of implant failure occurred in this area. Although quadrilateral plate fractures are not formally considered as a separate parameter of acetabular fracture classification, they are frequently encountered in various fracture patterns and gradually attract the attention of orthopedic surgeons and even considered to be an important factor affecting the complexity of surgery [5, 6].

Over the past decade, with the renovation of internal fixation technologies, various methods have been described to address this particular fracture fragment, including using a 1/3 tubular plate [7], T-shaped [8], or L-shaped plate [9] across the pelvic brim as a spring plate to buttress the medial wall or using a long infrapectineal buttress plate [10]. These internal fixation devices are not entirely without any limitations, and in most cases, it is extremely difficult to fix the quadrilateral plate especially in the absolute “dangerous zone” directly by screws and also exists a high risk of screws penetrating the hip by mistake. Although many scholars have proposed several methods such as inserting screws under direct vision after hip capsulotomy or increasing fluoroscopic to determine the location of screws, etc., the above methods will inevitably prolong the operation time and increase the risk of bleeding and infection [11, 12].

Since 2005, our department has innovatively proposed the method of dynamic anterior plate screw system for the quadrilateral area (DAPSQ) which was composed of a specially shaped reconstruction plate and several buttress screws of a quadrilateral plate (quadrilateral screws) to treat acetabular fractures involving quadrilateral plate. This fixation method has been patented in China (No. ZL 2013 2 0106378.0), and the long-term follow-up results have observed that it is an effective and safe choice for treatment of quadrilateral plate fractures [13]. However, the reconstruction plate of DAPSQ needs temporary shaping during operation. The proportion and torsion angle of each part depends on the experience of surgeons. It is difficult to form a unified standard and may also increase surgical time and intraoperative bleeding. In order to overcome the above problems, our team has performed many technical innovations and designed a set of standardized titanium plates of DAPSQ (No. ZL 2016 2 1494131.0) according to the acetabular anatomic parameters of the Chinese population.

The primary objectives of this study are to present surgical techniques of DAPSQ and compare the clinical efficacy of standardized titanium plate and the reconstruction plate of DAPSQ in the treatment of quadrilateral plate fractures. The secondary objectives are to compare the intraoperative conditions including surgical time, bleeding, and blood transfusion and postoperative complications between the two groups.

## Patients and methods

### Patients

After getting approval from our institution’s ethical committee (ethical review number: 2018024-1), we led a retrospective case-matched cohort study at our level 1 trauma center of the Military General Hospital between May 2016 and May 2018. Inclusion criteria were as follows: (1) all types of acetabular fractures involving the quadrilateral plate, (2) treated with standardized titanium plate of DAPSQ, (3) age > 18 years, and (4) fresh fractures. Exclusions included open or pathologic acetabular fractures, patients with pre-existing osteoarthritis of the affected hip. Written informed consent was obtained from all the patients.

Twenty-two consecutive acetabular fracture patients were treated with standardized titanium plate of DAPSQ (STP group) formed group 1. Then, the fractures in group 1 were matched according to age, gender, fracture pattern, and surgical approach to similar cases from our databases which included more than 140 cases treated with the reconstruction plate of DAPSQ from January 2008 to January 2016. This pool of cases was then used to randomly choose individual cases to create a 1:1 proportion, and this matched cohort was group 2 (RP group).

### Surgical technique

#### Preoperative management

The initial management of acetabular trauma followed the principles of the Advanced Trauma Life Support (ATLS), and the most important was to keep the stability of vital signs [14]. All injuries were evaluated preoperatively with radiographs and CT scans. Acetabular fractures were categorized according to the Judet and Letournel classification [2]. Skeletal traction was applied via the femoral condyles or tibial tubercle in all patients. One day before the operation, an autologous blood transfusion machine and heterogeneous blood (> 1000 mL) were prepared.

### Operation procedures

All fractures were treated by 2 senior orthopedic surgeons on a radiolucent table using a standard ilioinguinal approach described by Letournel [15] or combination with the Kocher-Langenbeck approach. Through the “second window” of the ilioinguinal approach, the acetabular anterior column, pelvic boundary, and the upper part of the quadrilateral plate could be directly exposed or touched. The first step was to reduce the medial dislocation of the femoral head, and the restoration of the continuity of the pelvic ring. Next, push quadrilateral plate fracture fragment into its bed until a smooth quadrilateral surface with no external step off was obtained.

#### STP group

**Fig. 1:**
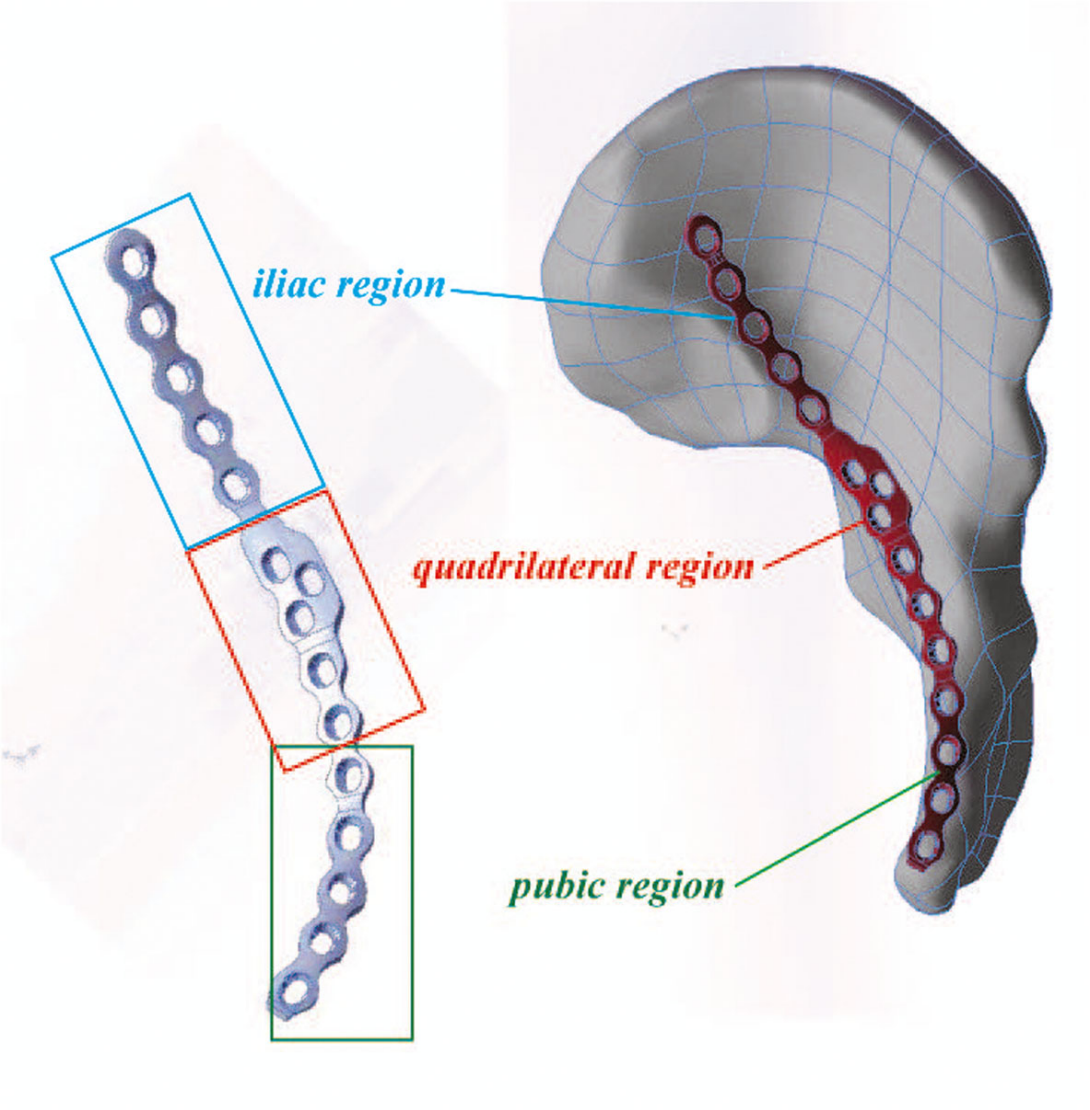
The structure and components of DAPSQ. DAPSQ was placed on the superior arcuate line, and the ends extended toward the iliac wing and the superior pubic ramus, respectively. According to the placement trajectory on the pelvis, the DAPSQ plate was divided into three parts: the iliac region, the quadrilateral region, and the pubic region. And the screws placed in the quadrilateral region were called “quadrilateral screws”

**Fig. 2:**
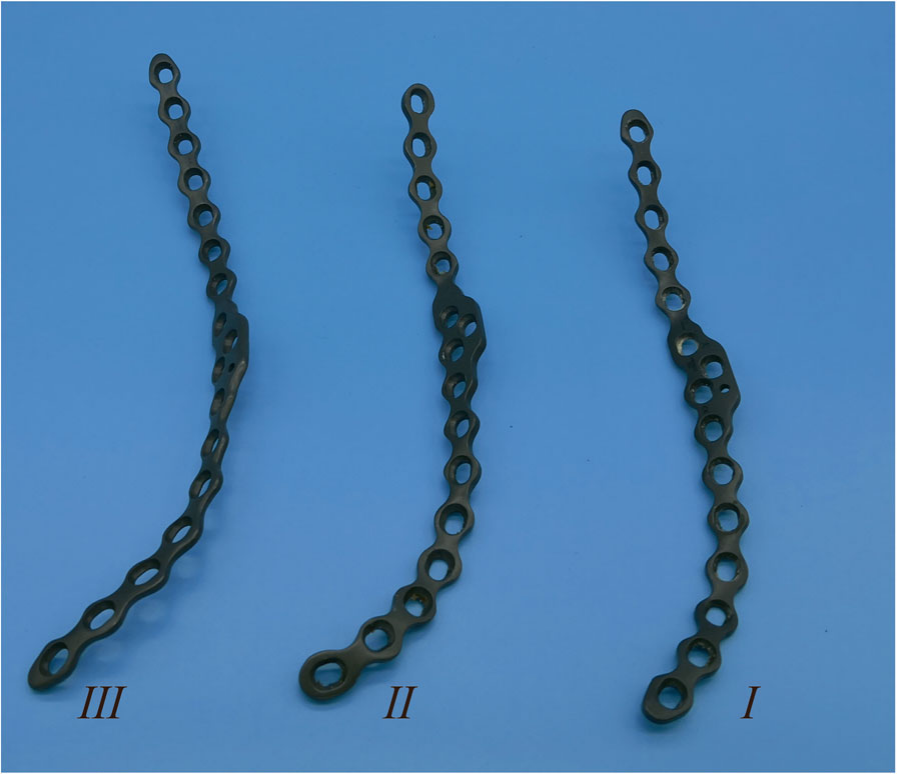
Three different models of the standardized titanium plate. According to the total anatomical length of the placement trajectory and the different proportion of the length in the three regions (the iliac region, the quadrilateral region, and the pubic region) measured by Chinese population, DAPSQ have been divided into three models: I, II, and III

**Fig. 3:**
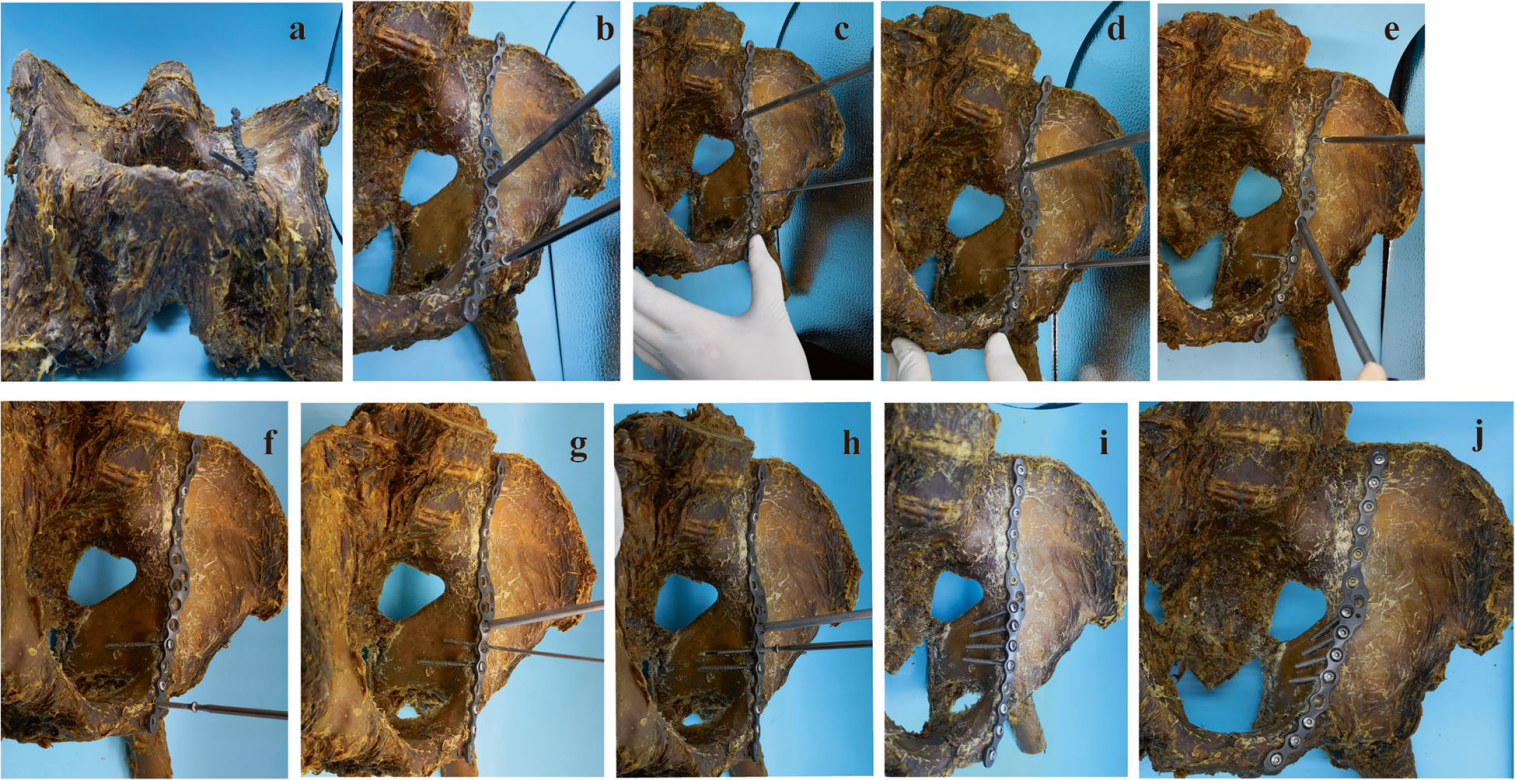
The placement sequence of screws. The cadaveric model (The Southern Medical University Anatomy Laboratory, Guangzhou, China) depicted the placement sequence of screws. **a** Choose an appropriate model of a titanium plate and both ends of the plate were upturned after placed. **b** The first screw was fixed on the pubic region. **c** Drill semi-“U” type holes parallel to the surface of the quadrilateral plate. **d** Insert the first quadrilateral screw along the pelvic brim and parallel to the surface of the quadrilateral plate, and only the 1/3 to 1/2 transverse diameter of the quadrilateral screw was screwed into the bone. **e** Then, move the plate of the iliac region away from the pelvic rim, so that the first quadrilateral screw can firmly attach to the bone surface, and insert a screw in the iliac region. **f** Subsequently, two fixation screws were inserted into the pubic and the iliac region, respectively, to stabilize the acetabular anterior column. **g**, **h** A 4.5-mm screwdriver was inserted into the adjacent screw hole to lift the internal edge of the plate, so that the 1/3 to 1/2 of the screws hole were exposed to the inner edge of the pelvic brim. Then, drill and insert the second quadrilateral screw. After the placement was completed, the screwdriver was removed, and the plate can naturally bounce back to provide the elastic retraction force for quadrilateral screws to block the inward displacement of the quadrilateral plate. **i**, **j** Place the residual screws in the iliac and the pubic region. And during the process of screw insertion, the titanium plate was gradually attached to the bone surface, and the torsion and elastic recoil of the plate could provide a strong holding force for quadrilateral screws to block the inward displacement of quadrilateral plate.

After the reduction was accomplished in the STP group, an appropriate model of the standardized titanium plate of DAPSQ was selected and placed on the superior arcuate line, and the ends extended toward the iliac wing and the superior pubic ramus (Fig. 1). The titanium plate was divided into three parts: the iliac region, the quadrilateral region, and the pubic region according to the placement trajectory on the pelvis (Fig. 1). We have preliminary designed three models based on the total anatomical length of the placement trajectory and the different proportions of the length in the three regions measured by the Chinese population (Fig. 2). Before screw insertion, both ends of the titanium plate were up-warped and not firmly attached to the bone surface. But during the screw insertion, the titanium plate could gradually firmly attach to the bone surface and detailed screw placement methods are showed in Fig. 3. The key surgical steps were as follows: two or more fixation screws on the iliac and pubic region should first be fixed to stabilize the acetabular anterior column. Then, with the help of a 4.5-mm screwdriver, quadrilateral screws were inserted along the pelvic brim and parallel to the surface of the quadrilateral plate under direct vision, and only the 1/3 to 1/2 transverse diameter of the quadrilateral screw was screwed into the bone to avoid penetrating the hip. And during the process of screw insertion, the torsion and elastic recoil of the plate could provide a strong holding force for quadrilateral screws to block the inward displacement of the quadrilateral plate. Also, make sure the distal end of the quadrilateral screws extended at least 10 mm beyond the fracture line.

#### RP group

**Fig. 4:**
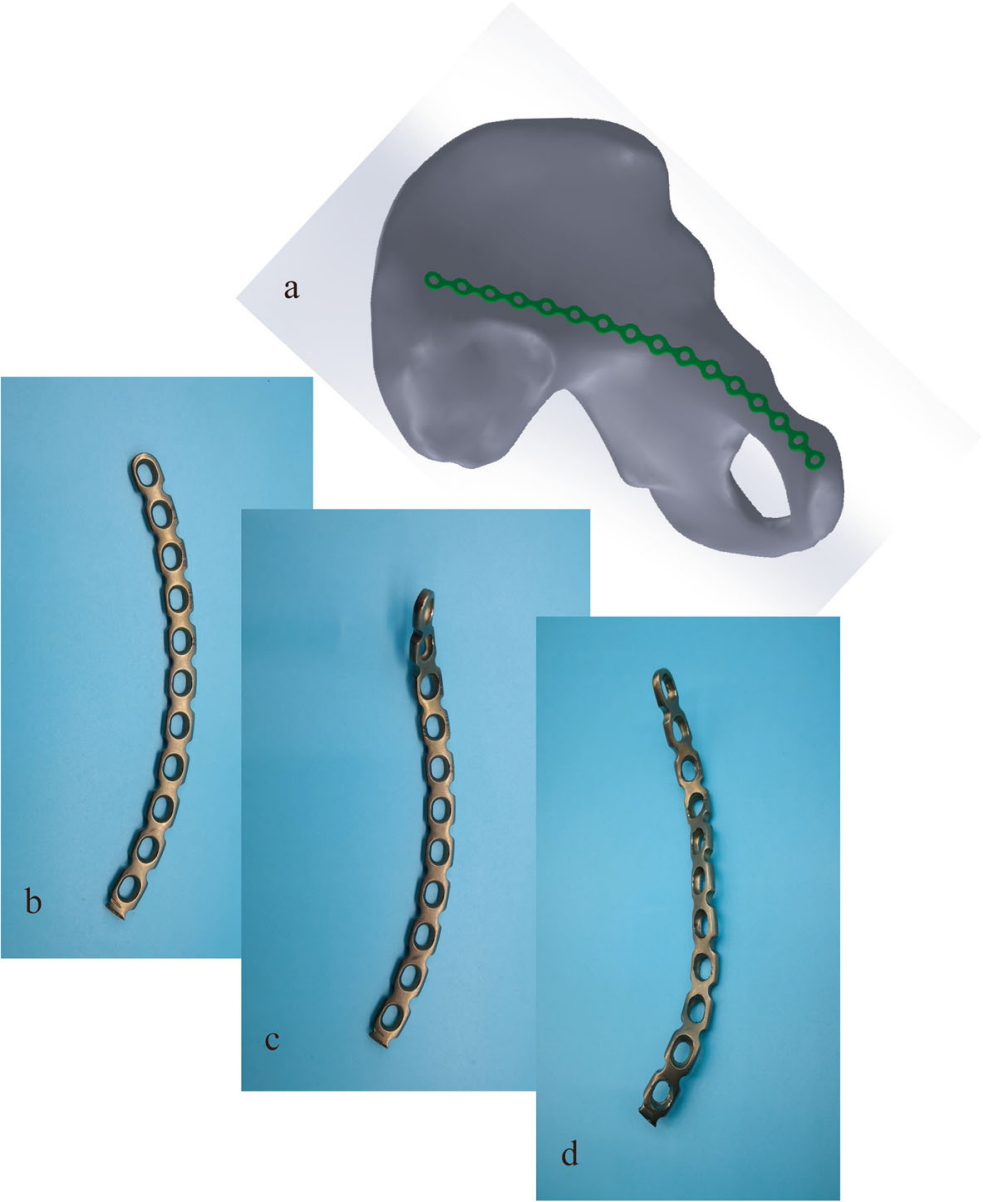
The shaping steps of the reconstruction plate. **a** First, the total anatomical length of the placement trajectory on the pelvis was measured. **b** Then, select an appropriate length of the arc-shaped reconstruction plate. **c** Both ends of the plate were reverse twisted and upturned. And the torsion angle of the iliac and pubic regions was higher than the radian of the bone surface, so both ends of the plate were upturned after placed. **d** The plate in the quadrilateral region slightly incline into the pelvis about 15°

A reconstruction plate was used in patients of the RP group. The shaping steps were as follows: first, select a 12- to 16-hole arc-shaped reconstruction plate based on the total anatomical length of the placement trajectory. Then, both ends of the plate were reverse twisted and upturned by using a bender and screwdriver. The torsion angle of the iliac and pubic regions was higher than the radian of the bone surface, so both ends of the plate were upturned after placed, and the plate in the quadrilateral region slightly inclines into the pelvis about 15° (Fig. 4). In order to achieve the best effect of quadrilateral buttress screws, the torsion angle and the proportion of the length in the three regions of the plate should be adjusted repeatedly. And the screw insertion methods were similar to the STP group.

In addition, if the reduction through a single ilioinguinal approach was not satisfactory or patients complicated with fractures of the acetabular posterior wall, adding the Kocher-Langenbeck approach was an appropriate choice. Throughout the application of DAPSQ, the obturator neurovascular bundle was identified and protected. Fracture reduction and implant positioning were carefully checked using fluoroscopy prior to closure of the wound.

### Postoperative management

Intravenously administered antibiotics were continued for 24 h. The drainage tube was removed within 3 days (24 h drainage flow < 20 mL). Patients started early rehabilitation after awakened from anesthesia and were instructed to non-weight-bearing exercises such as passive and active ipsilateral hip flexion or extension motion on the affected limb for 4–6 weeks. Then, protected weight-bearing exercises were encouraged till 8–12 weeks and gradually progress to full-weight bearing at 12 weeks.

### Method of assessment and data collection

Gender, age, fracture pattern, mechanism of injury, fracture side, concomitant injuries, the time between injury and surgery, surgical time, intraoperative bleeding, blood transfusion, and hospital stay time were all collected. Complications including the deep venous thrombosis, sciatic nerve problem, lateral femoral cutaneous nerve injury, surgical site infections, posttraumatic arthritis, heterotopic ossification, screws penetrating into the hip joint cavity, and implant failure were documented.

The immediate postoperative radiographs (anteroposterior of the pelvis and Judet views of the affected acetabulum) and 3D CT reconstruction were reviewed, and the reduction was evaluated using the Matta radiological criteria by 2 senior orthopedic surgeons [16]. The Matta grading scores were classified as anatomic (0–1 mm), imperfect (2–3 mm), or poor (> 3 mm) based on the maximal displacement on all views.

Regular outpatient review and follow-up were performed 1, 2, 3, 6, and 12 months after surgery and then yearly thereafter. Changes in clinical function, radiographic progress, fracture healing, and complications were recorded. Functional outcomes were evaluated by the modified Merle d’Aubigné score [17] assessed at the last follow-up and categorized as excellent (18 points), good (15–17 points), fair (13 or 14 points), or poor (< 13 points).

### Statistical analysis

Data was coded and analyzed with the statistical package SPSS version 19.0(IBM Corp, Armonk, NY). Continuous variables were expressed as mean ± standard deviations and categorical variables with absolute frequencies and percentages. Independent sample *t* test was used to compare quantitative variables. Chi-squared test or Fisher’s exact test was used to compare categorical variables. While the ranked data were analyzed with Mann-Whitney *U* rank sum test. Cohen’s Kappa Index was measured to estimate the inter-observer agreement. A value of *P* < 0.05 was considered statistically significant.

## Results

### Demographics

Twenty-two patients, including 18 males and 4 females with an average age of 46.7 years (range from 23 to 64 years) were treated with standardized titanium plate of DAPSQ in the inclusion period. According to the Letournel-Judet classification, there were 12 associated both column, 2 anterior column, 4 anterior column and posterior hemitransverse, 2 T-shaped, 1 transverse and posterior wall, and 1 transverse fracture. Randomization resulted in a matching ratio of 1:1 (Table 1).

**Table 1:** Fracture types and surgical approach in the standardized titanium plate (STP) group and reconstruction plate (RP) group

	STP group (*n* = 22)	RP group (*n* = 22)
Fracture type (*n*, %)		
Both column	12 (54.5%)	12 (54.5%)
Anterior column	2 (9.1%)	2 (9.1%)
ACPH	4 (18.2%)	4 (18.2%)
T type fracture	2 (9.1%)	2 (9.1%)
Transverse and posterior wall	1 (4.5%)	1 (4.5%)
Transverse fracture	1 (4.5%)	1 (4.5%)
Surgical approach (*n*, %)		
Ilioinguinal	17 (77.3%)	17 (77.3%)
Ilioinguinal+Kocher-Langenbeck	5 (22.7%)	5 (22.7%)

Patients were evaluated in terms of their demographic variables. There were no significant differences between the groups with regard to gender (*P* = 0.176), age (*P* = 0.202), mechanism of injury (*P* = 0.646), fracture side (*P* = 0.353), concomitant injuries (*P* = 0.918), and mean time between injury and surgery (*P* = 0.799). Patient demographics and characteristics are shown in Table 2.

**Table 2:** Comparison of the different variables between the STP and RP groups

Variable	STP group (*n* = 22)	RP group (*n* = 22)	*P* value
Gender, male (*n*, %)	18 (81.8%)	14 (63.6%)	0.176
Age, years (mean ± SD)	46.7 ± 11.6	50.8 ± 8.9	0.202
Mechanism of injury (*n*, %)			
Fall from height	11 (50%)	8 (36.4%)	0.646
Traffic accident	9 (40.9%)	11 (50%)	
Others	2 (9.1%)	3 (13.6%)	
Fracture side, left (*n*, %)	15 (68.2%)	12 (54.5%)	0.353
Concomitant injuries (*n*, %)			
Head trauma	5 (22.7%)	6 (27.3%)	0.918
Spine fracture	1 (4.5%)	2 (9.1%)	
Limb fracture	9 (40.9%)	6 (27.3%)	
Rib or clavicle fracture	4 (18.2%)	5 (22.7%)	
Dislocation of hip	3 (13.6%)	2 (9.1%)	
Others	3 (13.6%)	2 (9.1%)	
Time to surgery, days (mean ± SD)	8.8 ± 3.3	8.6 ± 2.6	0.799
Surgical time, min (mean ± SD)	245.1 ± 54.6	286.8 ± 59.3	**0.020**
Blood loss, mL (mean ± SD)	1136.4 ± 686.3	1777.3 ± 944.6	**0.014**
Blood transfusion, mL (mean ± SD)	780.9 ± 685.2	1256.8 ± 763.2	**0.035**
Hospital stay time, days (mean ± SD)	25.7 ± 6.3	26.8 ± 7.8	0.612
Quality of reduction (*n*, %)			
Anatomic (0–1 mm)	16 (72.7%)	14 (63.6%)	0.490
Imperfect (2–3 mm)	5 (22.7%)	6 (27.3%)	
Poor (> 3 mm)	1 (4.5%)	2 (9.1%)	

Bold entries indicate statistically significant *P* value

### Intraoperative conditions

All patients underwent surgery through a single ilioinguinal approach (77.3%) or combined with the Kocher-Langenbeck approach (22.7%). There were 2 to 4 quadrilateral screws used to control the medial displacement of the quadrilateral plate in all patients. For the RP group, a 14- or 16-hole reconstruction plate was most commonly used, while type I standardized titanium plate was most used in the STP group.

The mean surgical time was increased in the RP group (mean 286.8 min, range from 185 to 397 min) compared with the STP group (mean 245.1 min, range from 155 to 355 min) with a mean difference of 41.7 min (*P* = 0.020). Intraoperative mean blood loss and transfusion were significantly lower in the STP group compared with the RP group (bleeding 1136.4 vs. 1777.3 mL, *P* = 0.014; transfusion 780.9 vs. 1256.8 mL, *P* = 0.035). There was no significant difference in length of postoperative stay in both groups (*P* = 0.612). The mean postoperative stay was 26.8 days in the RP group vs 25.7 days in the STP group. Considering that the learning curve of the new technique may affect the results, all cases in the RP group were selected after 2008, and the technology has been used in our hospital for more than 3 years.

### Quality of reduction

The reduction quality of the acetabulum was evaluated by two independent senior orthopedic surgeons according to the Matta radiological criteria. And the degree of agreement between them was 84.1%, with a kappa value of 0.68, which indicated substantial agreement. Sixteen cases (72.7%) were graded as the anatomical reduction in the STP group, 5 cases (22.7%) as imperfect, and 1 case (4.5%) as poor. In the RP group, 14 patients (63.6%) showed an anatomical reduction, while 6 (27.3%) and 2 (9.1%) patients showed an imperfect and poor reduction, respectively. There was no statistically significant difference (*P* = 0.490) (Table 2). Postoperative 3D CT reconstruction and X-ray had shown that there was no one quadrilateral screw entering the hip and no one case occurring early fracture displacement or implant failure.

### Functional outcome

**Fig. 5:**
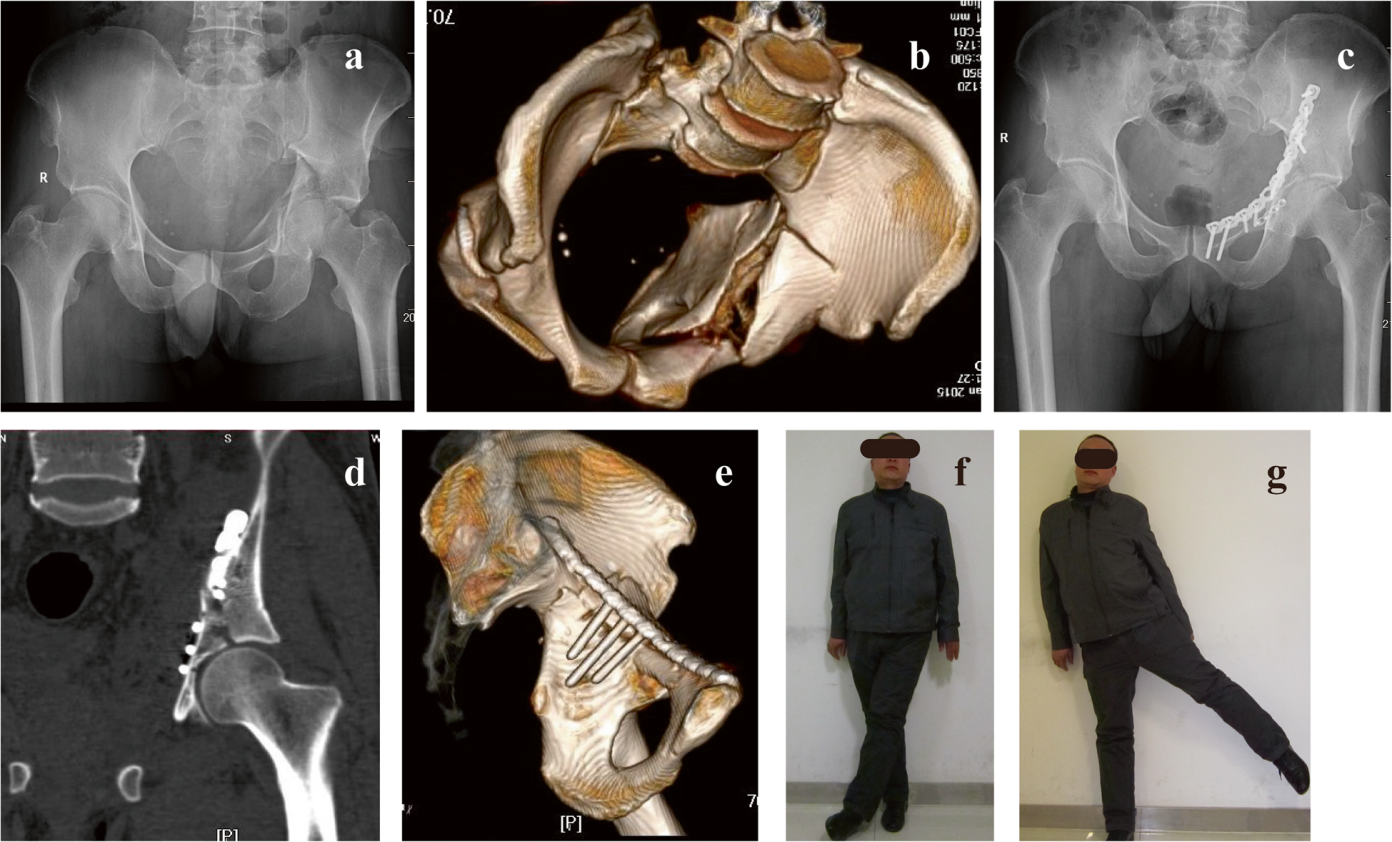
A typical case showing the use of the reconstruction plate for a displaced acetabular fracture. A 48-year-old man presented with both column fracture of the left acetabulum following a fall from a height. Preoperative AP (**a**) and 3D CT reconstruction (**b**) of the pelvis have confirmed the fracture pattern. On the 8th day after the injury, fixation was performed through the ilioinguinal approach by using a 12-hole reconstruction plate with 3 quadrilateral screws to control the medial displacement of the quadrilateral plate (**c**). Postoperative CT scanning image (**d**) and 3D CT reconstruction (**e**) showing the quadrilateral screws were located on the surface of the quadrilateral plate, and there was no one quadrilateral screw entering the hip joint cavity. At his 2 years of follow-up visit, the patient was symptom-free. The modified Merle d’Aubigné evaluation was scored as good (**f**, **g**)

**Fig. 6:**
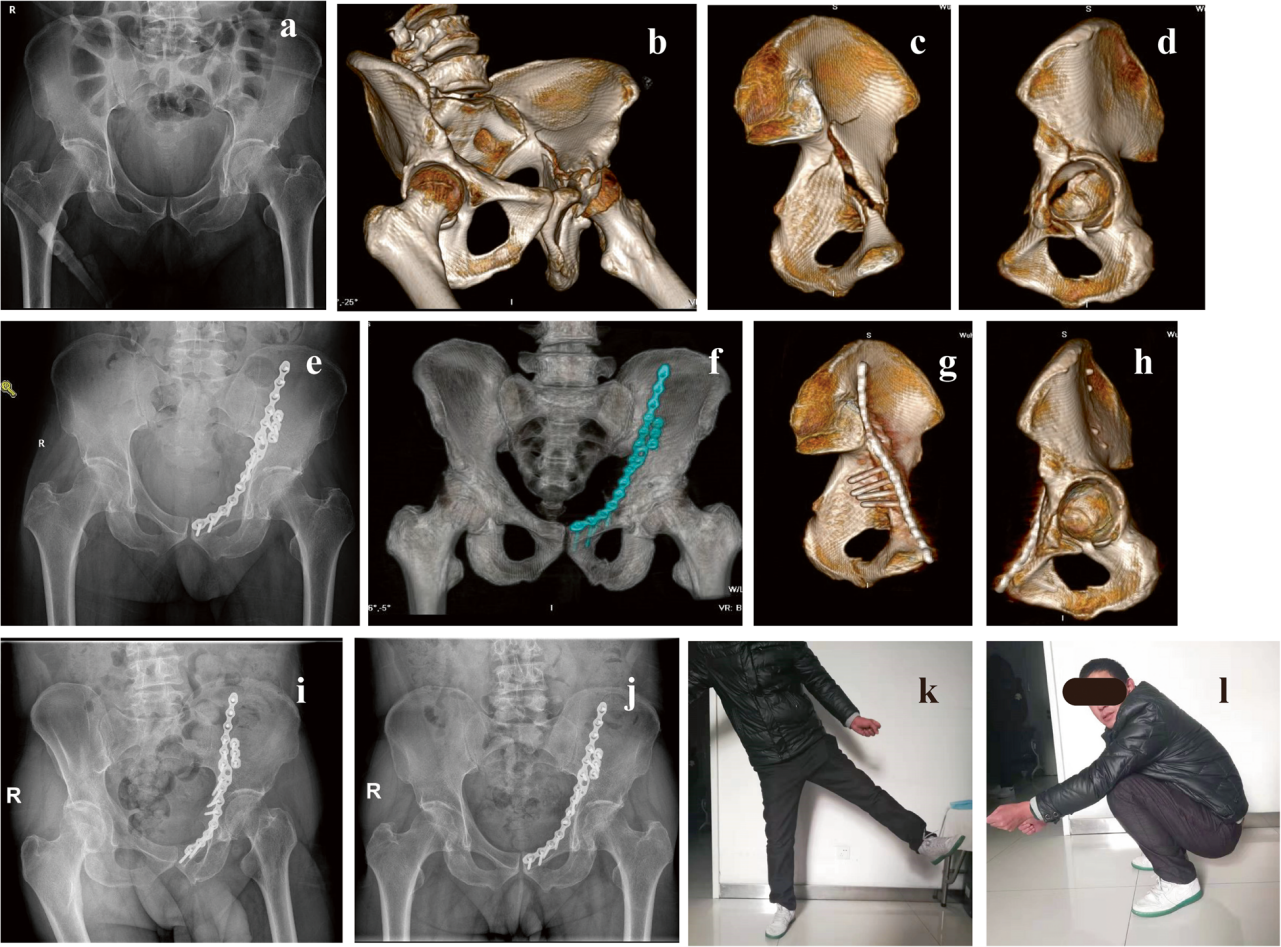
The second typical case showing the use of the standardized titanium plate for a complex acetabular fracture. A 56-year-old man presented with anterior column and posterior hemitransverse fracture of the left acetabulum following a traffic accident. Preoperative AP (**a**) and 3D CT reconstruction (**b**–**d**) of the pelvis have confirmed the fracture pattern. On the 9th day after the injury, fixation was performed through the ilioinguinal approach using the type I standardized titanium plate with 4 quadrilateral screws. Postoperative AP (**e**) and 3D CT reconstruction (**f**–**h**) showing Matta’s X-ray evaluation was scored as excellent. The AP (**i**) and Judet views (**j**) after 1 year showing a good radiological result and no evidence of osteoarthritic change. The modified Merle d’Aubigné evaluation was scored as excellent and the hip function of abduction (**k**) and flexion (**l**) was satisfied

The mean time of follow-up was 23.1 months in the STP group vs 26.1 months in the RP group (*P* = 0.260). According to the radiological evaluation at 12 weeks of follow-up, bony healing was achieved in all patients. At the last follow-up, the functional outcome in the STP group according to the modified Merle d’Aubigné score was excellent in 11 cases (50%), good in 9 cases (40.9%), fair in 1 case (4.5%), and poor in 1 case (4.5%). While the results in the RP group were excellent in 9 (40.9%), good in 9 (40.9%), and fair in 4 (18.2%). Although the excellent-to-good rate of the STP group was higher than the RP group (90.9% vs 81.8%), there was no significant difference between the two groups (*P* = 0.457). Detailed data was summarized in Table 3, and two typical cases were shown in Figs. 5 and 6.

**Table 3:** Postoperative outcome measurements

	STP group (*n* = 22)	RP group (*n* = 22)	*P* value
Mean time of follow-up, month (mean ± SD)	23.1 ± 8.9	26.1 ± 8.8	0.260
Modified Merle d’Aubigné score (*n*, %)			
Excellent	11 (50%)	9 (40.9%)	0.457
Good	9 (40.9%)	9 (40.9%)	
Fair	1 (4.5%)	4 (18.2%)	
Poor	1 (4.5%)	0	
Rate of excellent-to-good (%)	90.9%	81.8%

### Complications

The mean complication rate was 36.4% in the RP group which was higher compared with the STP group (18.2%), but there was no significant difference (*P* = 0.176). In the RP group, one patient presented with iliac vein thrombosis and pulmonary embolism, and a vena cava filter was inserted on the third day after the operation. Superficial wound infection was observed in 1 patient and was treated with antibiotics (2 weeks) and superficial wound debridement. Two cases of lateral femoral cutaneous nerve injury had recovered within 2 months. Four cases had developed into mild post-traumatic arthritis according to the Kellgren-Lawrence osteoarthritis classification system [18]. In addition, lateral femoral cutaneous nerve injury (1 case) and post-traumatic arthritis (2 cases) were also observed in the STP group, and one case finally required THA at 48 months. Sciatica symptoms developed in 1 patient and recovered after nerve nutrition drugs (vitamin B_12_) were applied. None of the patients in two groups had heterotopic ossification or loss of reduction at the last follow-up (Table 4).

## Discussion

The quadrilateral plate refers to the medial wall of the acetabulum and is not specifically considered as a parameter in most classification systems. Compared to the thick superior weight-bearing area, it is a bony structure that is relatively thin and requires less force to fracture it. When a high-energy injury occurs, the medial displacement of the femoral head often results in the fracture of the quadrilateral plate, even the central dislocation of the femoral head [8–10]. And the quadrilateral plate fractures are most frequently associated with both-column, anterior column, and posterior hemitransverse or T-shaped fractures. The current consensus on the treatment of acetabular fractures is to relieve pain, allow early mobilization, and restore function by restoring the normal hip anatomy. Previous studies have shown that the reduction of the quadrilateral plate fracture played an important role in the surgical results and failure to restore the quadrilateral plate with good reduction and reliable fixation would inevitably lead to a high incidence of complications such as joint dysfunction or post-traumatic arthritis [2–4]. As the presence of quadrilateral surface injury probably plays a decisive role in outcomes, optimal treatment of the quadrilateral fracture is very important. On the one hand, it is necessary to restore and coincide with the rotation center of the femoral head and the acetabulum. On the other hand, the fixation of the quadrilateral plate needs to overcome the inward and upward force of the femoral head. However, due to the deep location, weak bones, and being surrounded by many important blood vessels and nerves, quadrilateral plate fracture has always been a constant challenge to orthopedic surgeons [5, 6].

Treatment options for acetabular fractures include conservative and surgical methods. Over time, prognostic indicators of outcome have been defined and this has led to an increasing trend toward operative fixation. Currently, more and more operative fixation methods for quadrilateral plate fractures are being reported. The most common methods include anterior column reconstruction titanium plate combined with lag screw in posterior column [19, 20], reconstruction titanium plate of anterior and posterior column [21]. Letournel and Judet [2, 15] described the fixation method of long screws inserting along the pelvic brim and through the quadrilateral plate. They also commented that this technique was limited if the quadrilateral surface was comminuted or accompanied with severe osteoporosis. Although a biomechanical study by Shazar et al. [22] has shown that periarticular long screws could increase the stability of plate fixation, it cannot be ignored that this method can always withstand the high risk of hip penetration. In most cases, screw holes in the quadrilateral region of the plate are vacant without screws. If insertion is necessary, the direction of the screw should deviate from the joint surface, or a short screw less than 12 mm; otherwise, the screw may penetrate into the hip [10, 13]. Abandoning the direct fixation or screws away from the quadrilateral plate means the stability of the fixation will decrease [22]. Although the use of a lag screw in the posterior column improves the stability, it requires the high integrity of anterior and posterior column fragments [19, 20].

**Table 4:** Descriptive data of postoperative complications

	STP group (*n* = 22)	RP group (*n* = 22)	*P* value
Deep venous thrombosis	0	1	
Sciatic nerve problem	1	0	
Lateral femoral cutaneous nerve injury	1	2	
Superficial wound infection	0	1	
Posttraumatic arthritis	2	4	
THA	1	0	
Incidence of complication (*n*, %)	4 (18.2%)	8 (36.4%)	0.176

In order to avoid the screws mistakenly entering the hip, Mears et al. [7] first described the technique of “buttress plate” instead of periarticular screws. A one-third tubular plate used in this fashion was bent in an oblique manner and contoured over the pelvic brim to buttress the medial acetabular wall like a spring. However, subsequent studies have found that it was difficult to provide adequate medial buttress support for all individuals, and post-operative nursing of turning-body-over could affect the stability of the fixation [9, 23]. Then, Farid et al. [24] introduced a method of one-third tubular plate combined with cerclage wire to treat quadrilateral plate fractures. Although the fixation effect was improved, since the cerclage wire needed to pass through the greater sciatic notch, it was easy to injure the sciatic nerve and surrounding vessels. To date, newer buttress plate constructs that span the posterior and anterior columns through the quadrilateral surface have been developed. Among them, Stryker’s standardized titanium plate such as the infrapectineal or suprapectineal quadrilateral surface buttress plates are most researched. Vitro biomechanical studies have showed that standard buttress plate fixation of acetabular fracture resulted in at least as good and, in some cases, superior to traditional forms of fixation [21, 25]. But their clinical results still need further follow-up.

Unlike the above methods, the DAPSQ plate is a simple and ingenious design based on the anterior reconstructed titanium plates. The core idea of this fixation technology is elastic fixation and dynamic compression. First, quadrilateral screws were located on the bone surface and fixed under direct vision through the second window of the ilioinguinal approach. Only the 1/3 to 1/2 transverse diameter of the quadrilateral screw was screwed into the bone had avoided the risk of hip penetrating. Meanwhile, because the quadrilateral screws were not on the same plane as the screws fixed to the iliac and the pubic region of DAPSQ, the torsion and elastic recoil of the plate could provide a strong holding force for the quadrilateral screws to control the medial displacement of quadrilateral plate. Besides that, 2 to 4 quadrilateral screws parallel to the surface of the quadrilateral plate have formed a plane like the “bamboo raft.” More importantly, our team has evaluated the biomechanical stability of DAPSQ by the finite element analysis and cadaver model simulation [26, 27]. Finite element analysis results have shown that DAPSQ fixed acetabular double-column fractures had reliable biomechanical properties. Under 500 N load of standing, the Von-Mises stress was mainly concentrated across the titanium plate-screw junction near the large notch of the ischium and the maximum stress values were far less than the yield strength of titanium materials. Biomechanical test also showed that compared with anterior reconstruction titanium plate plus 1/3 tube buttress-plate, the stability of a double-column fracture model fixed by DAPSQ was superior. Our analysis suggests that the main reason may be that the fixation of 1/3 tubular plate only provides a single point support for the quadrilateral plate, and its biomechanical stability is obviously at a disadvantage compared to the “bamboo raft” multi-point elastic support formed by quadrilateral screws of DAPSQ. The biomechanical research conducted by Zha et al. [28] also has shown that compared with the quadrilateral spring plates of T-shaped, L-shaped, and H-shaped, the reconstruction plate combined with long screws had higher biomechanical stability for the treatment of quadrilateral plate fracture.

As the first propensity-matched cohort study comparing the use of standardized titanium plate and the reconstruction plate of DAPSQ in quadrilateral plate fractures, our data indicate that the application of standardized titanium plate has the advantages of a shorter operation time, less bleeding, and transfusion. These findings can be explained by the fact that repeated shaping steps are not required in the standardized titanium plate group, and an appropriate model can be individually selected during surgery. In addition, the process of screw placement is more standardized, and the corresponding matching apparatus is also designed and better utilized. One may argue that the reconstruction plate can provide a more reasonable design for all individuals, so that the reconstruction plate could firmly attach to the bone surface. However, the characteristics of this technique determine that it is not the key element for the success of fixation; on the contrary, repeated shaping steps may cause the elastic strength of the plate to decrease.

No significant differences were found between the two groups with regard to the quality of reduction using the Matta radiological criteria and the functional outcomes evaluated by the modified Merle d’Aubigné score. To our knowledge, only one retrospective study reported the similar technology of buttress screws to maintain the reduction of the quadrilateral fracture [29]. Their study included 35 quadrilateral plate fractures with an average age of 35 years (range 16–68 years). The anatomical reduction was achieved in 23 cases (66%), and the rate of excellent-to-good evaluated by the modified Merle d’Aubigné score was achieved in 31 cases (89%). Compared with our study, the difference may come from the different fracture patterns and surgical approaches. Although the technical characteristics of this study were similar to DAPSQ, that was, to control the medial displacement of the quadrilateral surface on the basis of avoiding the risk of hip penetrating, the fixation method is obviously different from DAPSQ. First, we shaped the reconstruction plate in a special way so that the plate did not fully conform to the bone surface before screw insertion. During the process of screw insertion, the torsional deformation of the DAPSQ plate could provide sufficient lateral dynamic compression for quadrilateral screws to block the medial displacement of the quadrilateral fracture. That is why we called the quadrilateral screws not only buttress screws, but also dynamic pressurized buttress screws. More importantly, we have drilled semi-“U” type holes parallel to the surface of the quadrilateral plate under direct vision, which not only enables the quadrilateral screws partially fixed the fracture fragments, but also make the screws firmly attach to the bone surface to prevent screws from loosening or withdrawing. The design of different types of standardized titanium plates also makes the selection of titanium plates more convenient during operation.

No significant differences were found between the two groups in the incidence of complications. As expected, no one had occurred the quadrilateral screws entering the hip. In clinical practice, we have found that DAPSQ standardized titanium plate has the following advantages over the reconstruction plate: (1) no additional shaping steps are required during operation, which greatly simplifies the procedure. (2) The pre-shaped design of the standardized titanium plate can provide more stable elastic strength for the quadrilateral screws and reduce the loss of elastic strength caused by the repeated shaping steps. (3) The process of screw placement is more standardized, and the corresponding matching apparatus is also designed and better utilized for standardized titanium plate.

This study presents some limitations: small sample size as well as a propensity-matched cohort study, where the study group originates from a prospective cohort and matched cases in a retrospective database, which may have selection bias. By matching the basic data as closely as possible (such as age, fracture type, and surgical approaches), selection bias can be minimized, but not eliminated. In addition, the most common type included in this study is double column fractures, which is different from the distribution of acetabular fractures in clinical practice. This selection bias is mainly due to our cases come from a level I trauma center where the most complex acetabular fractures are referred. Therefore, the results of this study need to be interpreted with caution and confirmed with large samples.

## Conclusion

DAPSQ is a simple and efficient technique for quadrilateral plate fractures and should be considered in the reconstruction of acetabular fractures. In terms of reduction quality and functional outcomes, standardized titanium plate, and reconstruction plate have similar results.

The standardized titanium plate of DAPSQ has the advantages of a short operation time, less intraoperative bleeding, and blood transfusion, and it is worth further promotion and research.

## Supplementary information

**Additional file 1:** The placement sequence of screws

## Data Availability

The datasets generated and/or analyzed during the current study are available from the corresponding author by reasonable request.

## Abbreviations

3D: Three-dimensional
ACPHT: Anterior column posterior hemitransverse type
AP: Anterior-posterior
CT: Computed tomography
DAPSQ: Dynamic anterior plate-screw system for quadrilateral plate
LFCNI: Lateral femoral cutaneous nerve injury
THA: Total hip arthroplasty
